# The Influence of Contextual Factors on the Process of Formulating Strategies to Improve the Adoption of Care Manager Activities by Primary Care Nurses

**DOI:** 10.5334/ijic.5556

**Published:** 2021-05-19

**Authors:** Ariane Girard, Pasquale Roberge, dith Ellefsen, Jolle Bernard-Hamel, Jean-Daniel Carrier, Catherine Hudon

**Affiliations:** 1 Facult de mdecine et de sciences de la sant, cole des sciences infirmires, Universit de Sherbrooke, Qubec, Canada; 2 Facult de mdecine et des sciences de la sant, Dpartement de mdecine de famille et de mdecine durgence, Universit de Sherbrooke, Qubec, Canada; Centre de recherche du centre hospitalier universitaire de Sherbrooke, Canada; 3 Facult de mdecine et des sciences de la sant, Dpartement de psychiatrie, Universit de Sherbrooke, Qubec, Canada

**Keywords:** implementation strategies, implementation planning process, care manager, collaborative care, integrated care, primary care nurse

## Abstract

**Background:**

Primary care nurses are well-suited to provide care management for common mental disorders, but their practices depend on context. Various strategies can be considered to improve the adoption of nursing care manager activities, but data from implementation studies rarely address strategy formulation.

**Aim:**

To analyze the influence of contextual factors on strategy formulation to improve the adoption of care manager activities by primary care nurses.

**Method:**

A qualitative multiple case study in three primary care clinics was carried out. Data were collected through individual interviews (n = 32) and observations (n = 7), working group meetings, and relevant documents. Thematic analysis was conducted.

**Results:**

Contextual factors influenced strategy formulation through organizational readiness for change, which resulted from tension for change and perceived organizational ability to implement change. Tension for change was generated through the perceived gap between patient needs and service availability, perceived compatibility with the nurses work environment, and their assessment of their capacity to perform care manager activities or acquire the necessary skills.

**Conclusion:**

Future studies should give sufficient attention to implementation strategy formulation and consider the dynamic role of organizational readiness for change when facilitating the adoption of evidence-based practices for common mental disorders in primary care.

## Background

In Canada, anxiety and depressive disorders are among the top five most frequently diagnosed long-term conditions [1]. These common mental disorders (CMDs) are strongly associated with long-term physical conditions such as diabetes or cardiovascular diseases [2]. The co-occurrence of mental and physical health conditions contributes to morbidity and disability, decreases quality of life [3,4], and increases health services utilization [2,5].

The collaborative care model (CCM), built on Wagners Chronic Care Model, has been developed to improve care quality for people with CMDs in primary care settings [6]. The CCM involves a team of health care professionals minimally including a general practitioner (GP), a care manager, and a mental health specialist, working together to implement an individualized plan developed by integrating scientific evidence with patient needs and preferences [7]. The CCM has shown convincing potential to cost-effectively improve the mental health status of people with long-term physical conditions [8,9,10].

The role of care manager, a key component of the CCM, consists in delivering and coordinating physical and mental health care and services for an assigned caseload of patients [11]. Primary care nurses (PCNs) often successfully act as care manager in published CCM studies [12,13,14]. Indeed, routine PCN activities have considerable overlap with care manager activities: e.g., mental health status assessment, monitoring the efficacy and side effects of prescribed medications, adjusting treatments in collaboration with the GP, and providing psychoeducation [11,15]. Despite this overlap, CCM implementation teams have faced many challenges when promoting care manager activities to PCNs, who often felt they did not have sufficient skills and knowledge to manage mental health-related care competently [16,17]. PCNs also reported contextual barriers to the adoption of care manager activities such as lack of collaboration between team members, excessive workload, or competing priorities from the leadership [17]. On the other hand, contextual facilitators to the adoption of care manager activities include leadership interest in mental health and technical support from an implementation team [18,19].

Given the influence of context on the adoption of care manager activities, strategies to facilitate their implementation should be carefully selected to fit local needs. Implementation strategies can be defined as methods or techniques used to enhance the adoption, implementation, and sustainability of a clinical program or practice [20, p. 2]. While the evidence is scarce on properly formulate strategies to implement the CCM and promote the adoption of care manager activities, stakeholders who are expected to change their practices should be involved in this process [18,19,21]. Implementation strategies are usually formulated during the planning or pre-implementation stage of the implementation process [22], which has been argued to receive insufficient attention in light of its crucial importance [23,24,25,26]. Indeed, the implementation planning stage is an opportunity to work with stakeholders to develop a proper understanding of the most significant determinants of change that should be accounted for in formulating contextually appropriate implementation strategies [22]. Moreover, implementation planning remains a stage of the implementation process itself, which means that contextual factors will also influence it. Therefore, lack of data on the influence of contextual factors on planning activities such as strategy formulation creates a blind spot in current knowledge on CCM implementation and the adoption of care manager activities by PCNs.

## Aim

We aimed to analyze the influence of contextual factors on the process of formulating strategies to improve the adoption of care manager activities by primary care nurses.

## Study setting

The study was conducted in family medicine groups (FMGs), the main primary health care organizational model in Quebec, Canada. FMGs are organizations led by a group of GPs working in collaboration with PCNs and social workers, and in some cases with other health care professionals such as pharmacists, nutritionists, or psychologists [27]. In FMGs, each GP is responsible for a caseload of patients for whom they share responsibilities with the other primary care teams members. PCNs role in FMGs is to provide long-term care for people with complex needs and provide acute care for a range of clienteles (including children, adolescents, adults, and the elderly) [28].

In Quebec, public sector health care and social services are under the responsibility of a regional health center. While GPs have a direct relationship with the provincial single-payer health insurance provider, nurses and other health care professionals working in FMGs are employees of the regional health center, which is responsible for ensuring the quality of professional practices. The medical director of each FMG is responsible for PCNs and other health care professionals work environment (e.g., facilities, equipment).

## Methodology

### Research design

We conducted a qualitative multiple case study in three FMGs [29,30] using an integrated knowledge translation approach to formulate implementation strategies with stakeholders. Integrated knowledge translation can be defined as an ongoing relationship between researchers and decision-makers (clinicians, managers, policy-makers, etc.) for the purpose of engaging in a mutually beneficial research project or program of research to support decision-making [31, p. 1]. To operationalize integrated knowledge translation in the planning stage of the implementation process, we followed steps one to four and part of step five of Grol & Wensings implementation of change model: 1) Development of a proposal for change; 2) Analysis of actual performance; 3) Problem analysis of target group and setting; 4) Development and selection of strategies; and 5) Development, testing and execution of an implementation plan [32]. We did not perform the two final steps of this model, i.e., integration of changes in routine care and continuous evaluation and adaptation of the plan.

We adopted a multiple case study methodology to reach the deep understanding of the context necessary to reach our aim [33]. We defined cases as the implementation planning process in each of the participating FMGs. Units of analysis within each case were: (1) current nursing and collaborative care practices for people with CMDs and long-term physical conditions and (2) the formulation of implementation strategies to optimize the adoption of care manager activities by PCNs.

We conducted the integrated knowledge transfer approach with regional and local stakeholders involvement, including an advisory committee, local working groups, clinicians, and patients. The advisory committees role was to counsel the research team regarding the provincial and regional contexts and support local working groups decision-making within FMGs. This committee included two patient partners with a history of CMD and at least one physical long-term condition, three researchers in the fields of collaborative mental health care and nursing, one nurse manager, one GP, one PCN, one psychiatrist, and one psychologist. Ethical approval was provided by the *Centre intgr universitaire de sant et de services sociaux de lEstrie-Centre hospitalier universitaire de Sherbrooke* (CIUSSS-CHUS) ethics committee.

### Recruitment

We recruited three FMGs based on convenience sampling and the lead GPs level of interest in this study. We contacted lead GPs by e-mail, providing a one-page summary of the project and describing the studys aim and the expected staff involvement and timeline. We chose to target three FMGs to improve the results transferability to similar contexts while maintaining the studys feasibility [30]. All three participating FMGs were located in medium-sized urban areas, but they varied in terms of mental health practices and resources (e.g., access to a consulting psychiatrist).

Within each FMG, a local working group was created, including at least one PCN and one GP. Local working groups were involved in discussing the gap between their practices and the CCM and in strategy formulation. In general, working group members were administrative leaders in their FMG or professionals whom their colleagues identified as mental health experts. Local working group members provided signed informed consent to participate in this study.

In each FMG, health care professionals involved in the care of people with CMDs (i.e., PCNs, GPs, psychologists, and social workers) were invited to participate in interviews. For patients recruitment, we asked participating PCNs to identify one adult patient diagnosed with at least one long-term physical condition and a depressive or anxiety disorder. Identified patients were then individually contacted by a research team member for participation in a qualitative interview and an observation session of a clinical encounter with their PCN. All participants to interviews and/or observation sessions provided signed informed consent before data collection.

### Data collection

Data collection was carried out throughout the implementation planning process. From December 2018 to April 2019, data were collected through the following sources: (a) face-to-face interviews (45 to 60 minutes) with patients and clinicians (n = 32); (b) non-participatory observations of a nurse-patient encounter (n = 7); and (c) relevant documents on professional activities and collaborative care (documents on specific programs for people with long-term physical conditions or CMDs, educational materials, clinical nursing mental health assessment guides, and health care professionals referral forms). In addition to interviews and observations, each participant completed a questionnaire. Patients were asked sociodemographic and health-related questions, and clinicians were asked questions relating to their professional role. In addition, every advisory committee and local working group meeting was summarized in a document including attendees identity and the main points discussed (January 2019 to January 2020).

We developed an interview guide for each type of participants (patients, PCNs, other primary care providers) to investigate current practices, challenges to nursing and collaborative care practices, and perceptions regarding an eventual change in practices. Interview guides were developed based on the results of previous studies on challenges to nursing and collaborative care practices for people with CMDs and long-term physical conditions in primary care clinics [15,34], and studies on barriers and enablers to implementing the CCM [17,18,19]. We reviewed the three interview guides respectively with a patient partner, a PCN, and a psychiatrist who were not otherwise involved in the study. The first author conducted interviews and observations and took field notes after each data collection session to record first impressions and propose links to previous interviews/observations. Interviews and observation sessions were audio-recorded and transcribed verbatim. Two additional researchers (EE, PR) also listened to two to three recordings to validate whether interviews captured all relevant elements of the practice and context. Finally, the first meeting with each local working group was audio-recorded while a professional research assistant took additional notes.

### Implementation planning process

To operationalize the implementation planning process, we used CCM implementation tools available free-of-charge on the University of Washington Advancing Integrated Mental Health Solutions (AIMS) Center website. Thus, two implementation tools were adapted with permission from the AIMS Center: (1) Patient-Centered Integrated Behavioral Health Care Principles & Task Checklist, and (2) CoCM Behavioral Health Care Manager: Sample Job Description, Typical Workload & Resource Requirements [11,35]. From those implementation tools, we constructed analysis tables to assess and compare each FMGs nursing and collaborative care activities with professional activities described in the CCM (see Additional files 12). ***Table 1*** presents the implementation planning process as conducted based on the first steps of the Grol & Wensing implementation of change model.

**Table 1:** Description of the implementation planning process. * For the purpose of the study, the term analysis of actual practices has been used instead of analysis of actual performance (per Grol & Wensings model) to better reflect our qualitative perspective. PCN = primary care nurse, GP = general practitioner, FMG = family medicine group, CCM = collaborative care model.


STEPS	GOAL	SUMMARY OF MAIN ACTIVITIES	PERIOD

**1) Proposal for change**	Carefully plan the change in practices and engage people directly involved	Conduct a scoping review on the role of the care manager [15] Develop tools to analyze practices Organize meetings with stakeholders: Nurse managers from the regional health center (n = 2): (1) present a proposal for change based on previous studies;(2) confirm their interest in changing practices and identify potential FMGs for recruitment The lead GPs and interested professionals in each FMG to present the project (n = 1 to 2) Members of the advisory committee to share current evidence on collaborative care and to discuss the feasibility of improving the role of PCNs through care manager activities (n = 1)	Jan. 2017Jan. 2019

2) Analysis of actual practices*	Fully understand current nursing and collaborative careactivities for people with CMDs and long-term physical conditionsto identify areas for improvement	Collect data on current practices(interviews, observations, documents) Describe actual practices (main activities, environment, collaboration, etc.) Schematize the collaborative care process in each FMG Compareand qualitatively assess care manager and other professional activities involvedin the CCM using two analysis tables	Dec. 2018Apr. 2019

**3) Problem analysis**	Identify determinants of practice that can be targeted and formulate potential strategies to improve PCNs care manager activities	Compare results of individual FMGs to visualize areas for improvement and identify setting-specific characteristics List the determinants of collaborative care and care manager activities by PCNs Conduct a meeting with the advisory committee (90 minutes) to clarify the problem and to explore potential strategies to improve PCNs care manager activities Conduct a meeting with each FMGs local working group (90 minutes) to validate results from practice analysis, discuss contextual challenges, formulate potential strategies for improvement, and assess professionals willingness to implement change in nursing care manager activities	Jan. 2019May 2019

**4) Selection of strategies and development of a plan**	Clarify the problem with primary care providers directly affected by the change of practice, select appropriate strategies tailored to local needs and develop an implementation plan	Conduct additional meetingswith local working groups to prioritize strategies and develop theimplementation plan (number and format of meetings varied between FMGs)	June 2019Jan. 2020


### Data analysis

We first conducted an intra-case analysis, followed by inter-case analysis [30,36]. For the intra-case analysis, meetings with local working groups served to build a case history regarding the formulation of implementation strategies. A systematic list of specifications was used to describe the case history for each FMG: the number of meetings conducted, the goal and attendance of each meeting, the main problem identified, stakeholders main concerns regarding the change in practices, and a summary of the implementation plan. We first conducted a thematic analysis of the case histories with an inductive approach, leading to the emergence of themes understood to have influenced the formulation of strategies to promote the adoption of care manager activities by PCNs. Field notes and results from the analysis of current practices were used to validate and enrich the initial case histories. Thereafter, we related the emerging themes to the contextual factors represented in the Consolidated Framework for Implementation Research (CFIR) [25]. One author (JBH) performed a thematic analysis for every transcribed document using the NVivo software. The interviewer (AG) reviewed the whole analysis process for each document, and both researchers discussed the main emerging themes in relation to the case histories until there were no remaining discrepancies in the interpretation of the CFIRs contextual factors involved. For each FMG, we also constructed a schematic representation of the processes leading to care manager activities by PCNs to highlight the logical impact of specific contextual factors on those processes.

Regarding inter-case analysis, we created matrices to explore differences and similarities among emerging themes across FMGs. We also synthesized the relationship between contextual factors and the formulation of strategies to promote the adoption of care manager activities in a schematic form. The results interpretation was verified throughout the analysis through peer validation involving all the authors and by comparison with published theories and models. In particular, Weiners theory on organizational readiness for change was instrumental in clarifying the links between contextual factors and the process of formulating implementation strategies [37].

## Results

### Characteristics of recruited FMGs and participants profile

***Table 2*** summarizes the main characteristics of each FMG. FMG01 and FMG03 were both university-affiliated, with a mandate and additional staff to train family medicine residents and primary care nurse practitioners. Nurses involvement in decision-making regarding their practices differed between FMGs. In FMG01 and FMG02, PCNs attended monthly team meetings to discuss their practices and to propose quality improvement projects to the medical director. In FMG03, PCNs met two to three times a year with their manager from the regional health center to discuss their practices and receive information about upcoming changes or programs.

**Table 2:** Main characteristics of FMGs.


	FMG01	FMG02	FMG03

Years since its creation	15	16	12

Number of sites	1	1	2

Number of patients registered (~)	30,00035,000	30,00035,000	10,00015,000

**TYPE AND NUMBER OF PROFESSIONALS**			

General practitioners	2530	3035	1015

Primary care nurses	6	5	3

Nurse practitioners	3	0	2

Social workers	3	3	1

Psychologist	1	1	1*

Pharmacists	2	1	1


* The psychologist in FMG03 had a teaching mandate rather than providing direct care to patients.

***Table 3*** summarizes the profile of study participants. In addition to the members of each local working group, a total of 33 participants were recruited for interviews and/or observations (25 clinicians and eight patients), only five of which were male (two clinicians, three patients). Working group members could also participate in interviews and/or observations. Detailed information on each clinician (gender, age, years of experience) and patient (gender, age, mental and physical conditions diagnoses) are reported in a related paper [38].

**Table 3:** Participants profile.


LOCAL WORKING GROUP MEMBERS			

TYPE OF PARTICIPANTS	FMG01	FMG02	FMG03

**Clinicians**	**N = 4**	**N = 3**	**N = 7**

	1 PCN with expertise in mental health 1 GP with expertise in mental health (lead GP) 1 nurse practitioner 1 nurse manager from the regional health center	1 PCN (leader) 1 GP with expertise in mental health (lead GP) 1 nurse manager from the regional health center (same as FMG01)	2 PCNs 1 quality improvement agent 2 GPs (including the lead GP) 1 social worker 1 psychologist

**INTERVIEW AND/OR OBSERVATION PARTICIPANTS**			

**Clinicians**	**N = 9**	**N = 8**	**N = 7**

	5 PCNs 1 GP 1 social worker 1 nurse practitioner 1 pharmacist	5 PCNs 1 GP 1 social worker 1 psychologist	3 PCNs 2 GPs 1 social worker 1 nurse practitioner 1 psychologist

Patients*	**N = 3**	**N = 3**	**N = 2**

	Had two or more long-term physical conditions (e.g., hypertension, diabetes, cholesterol). Two reported both substance use disorder and depression, one an anxiety disorder.	Had at least two long-term physical conditions. Two reported both anxiety and depressive disorders, one an anxiety disorder.	Had at least two long-term physical conditions. Both reported comorbid substance use, depressive, and anxiety disorders.


PCN = primary care nurse, GP = general practitioner. * Patients reported physical and mental health conditions in a questionnaire adapted from a validated French version of the disease burden morbidity assessment questionnaire [39,40].

### Case descriptions

#### Nursing and collaborative care practices

Collaborative care and nursing activities for people with CMDs varied across FMGs, leading to differences in the potential areas for practice improvement. For instance, PCNs in FMG02 had developed skills in mental health status assessment and used validated tools to measure anxiety and depressive symptoms (Patient Health Questionnaire-9 [PHQ-9], generalized anxiety disorder-7 [GAD-7]). Over the previous decade, FMG02 had developed an internal care pathway for screening, assessing, and managing the care of patients with mental health conditions, with PCNs being responsible for evaluating patients needs and establishing short-term recovery objectives with them.

All FMGs faced some issues regarding access to external mental health care services such as psychotherapy, psychiatric evaluations, or psychosocial support, resulting in waiting lists for services provided by the regional health center. In the three participating FMGs, patients also had to be put on a waiting list to receive the internal psychosocial teams services. Regarding specialized mental health care, the psychologist in FMG02 remarked that high demand and time constraints limited their capacity to deliver psychotherapy. FMG01 had access to two psychiatrists visiting the clinic part-time for consultations on GPs request, while GPs in FMG02 and FMG03 had to send psychiatric evaluation requests to the regional health center. In either case, PCNs had little interaction with psychiatrists, if at all. We report results from the analysis of actual practices in another manuscript [38].

#### Strategy formulation

Strategy formulation refers to the process of analyzing the problem and selecting and prioritizing implementation strategies with stakeholders. During strategy formulation, we invited stakeholders to consider potential issues with the adoption of care manager activities by PCNs, which led to the identification of significant contextual factors that they would take into account. ***Table 4*** presents contextual factors that each FMGs working group explicitly considered relevant to implementing change in their context. Contextual factors are labeled according to their CFIR definitions [25].

**Table 4:** Contextual factors taken into account when formulating strategies.


FMG01	FMG02	FMG03

OUTER SETTING		

External mental health service offers and patient needs*		

Patients expectation of close monitoring of their condition by a competent professional whom they trust and can refer to when dealing with mental health problems General difficulty accessing non-pharmacological treatments and services for CMDs		

**INNER SETTING**		

***Gapsbetween current practices and care manager activities***		

Varying degree of PCN involvement in the continuum of care and services for people with CMDs Lack of collaboration between GPs, NPs, and PCNs for the management of CMDs Lack of a clear definition of the role of PCNs for people with CMDs	Limitation of PCNs to short-term involvement in the management of CMDs, or to medication and health status monitoring when providing follow-up No tangible description regarding the clinics actual procedure/care trajectories and the role of PCNs for people with CMDs and long-term physical conditions	Lack of collaboration between PCNs and GPs in the management of CMDs General lack of collaboration between PCNs, GPs, and SWs Lack of a clear definition of the role of PCNs in general PCNs were not involved in the detection of anxiety and depressive symptoms in people with long-term physical conditions

***Access to knowledge and information***		

Uncertainty whether PCNs were comfortable enough and had sufficient knowledge to provide care manager activities for people with CMDs to implement changes in their practices	Uncertainty among working group members about how PCNs can be involved in psychosocial interventions	Uncertainty among PCNs about the feasibly of integrating care manager activities into their current workload (had to follow several chronic disease monitoring protocols for various clienteles) Lack of awareness among GPs and PCNs about an existing internal care protocol for depression, which the medical team had not approved Lack of training among PCNs to implement the existing depression care protocol

***Available resources***		

Not reported	Low nurses-to-physicians ratio (5 to 25) limiting PCNs ability to collectively care for the population of patients with anxiety and/or depressive disorders	Unstable roster of PCNs (maternity leaves, the arrival of new nurses)

***Compatibility***		

Not reported	Uncertainty with the respective role and responsibilities of PCNs and SWs regarding psychosocial interventions and follow-up	Not reported

***Relative priority***		

Not reported	Uncertainty whether adopting care manager activities was a priority for PCNs not on the working group	Uncerainty whether providing care manager activities to patients with CMDs was a perceived need for the entire medical team and nurses


CCM = collaborative care model, PCN = primary care nurse, GP = general practitioner, FMG = family medicine group, SW = social worker, CMD = common mental disorder. * Emerged from patients interviews in the three FMGs and from patient partners in the advisory committee, shared by the first authors during local working groups meetings.

The process of formulating strategies differed among FMGs. In FMG01, strategies evoked during problem analysis were relatively similar to those that were ultimately prioritized. In FMG02, strategies selected during problem analysis were completely revised as further inquiry revealed that PCNs were not interested in changing their current practice, but they felt that it might be warranted to benchmark their current practice for future reference when hiring new nurses. In FMG03, a change in the management leadership occurred between problem analysis and the final selection of strategies for implementation. As the new lead manager was involved in priorizing implementation strategies, they prioritized to improving nursing care manager activities for people with CMDs and long-term physical conditions. All strategies mentioned by working group members during either problem analysis or strategy selection are presented in ***Table 5***. We report strategies with guidance from the Expert Recommendations for Implementing of Change study regarding strategy definitions [41] and clusters [42].

**Table 5:** Formulation of strategies to improve the adoption of care manager activities by PCNs.


	FMG01	FMG02	FMG03

**Train and educate stakeholders**	Conduct educational meetings to train PCNs on care manager activities for people with CMDs Shadow other experts to offer clinical support to PCNs (potentially including coaching and case discussions with NPs) Make training dynamic by involving NPs Screen current training programs and develop or adapt educational materials with the regional health center **(P)**	Conduct educational meetings to inform PCNs on existing self-management support tools and on low-intensity psychosocial interventions that they can provide as part of a follow-up Develop educational materials in collaboration with the research team to facilitate the training of newly hired nurses in the clinics care trajectories and the role of PCNs for people with CMDs and long-term physical conditions **(P)**	Conduct educational meetings to train PCNs in screening anxiety and depressive symptoms **(P)**

**Support clinicians**	Revise PCNs professional role and responsibilities regarding care for CMDs Develop resources sharing agreements between the FMG and regional health center to ensure that PCNs have time available for training **(P)**	Revise PCNs professional role and responsibilities regarding the follow-up of people with anxiety or depressive disorders and clarifying the complementarity of the SW and PCN roles	Revise PCNs professional role in the follow-up of people with CMDs and long-term physical conditions **(P)** Remind PCNs managing long-term physical conditions of their role in screening for anxiety and depressive symptoms by adding a section on this topic in clinical protocols **(P)**

**Develop stakeholder interrelationships**	Obtain formal commitment from all PCNs to ensure readiness to change their practices **(P)** Capture local knowledge from FMG02 by consulting PCNs about their current practice for people with mental health problems **(P)**	Obtain formal commitment from all PCNs to ensure readiness to change their practices and consult them on strategies to prioritize for implementation **(P)**	Conduct local consensus discussion to evaluate the feasibility of optimizing the role of PCNs in providing care manager activities for people with CMDs and long-term physical conditions and to improve collaboration between PCNs, GPs, and SW during clinical follow-up Use a workgroup to clarify the role of PCNs and the local care trajectory for people with CMDs **(P)**

**Use evaluative and iterative strategies**	Conduct small trials cyclically with some GPs to test the implementation of change **(P)**		


CMD = common mental disorder, GP = general practioner, PCN = primary care nurses, NP = nurse practitioner, SW = social worker. (P) indicates strategies that were prioritized for implementation.

### Influence of contextual factors on the formulation of strategies

Throughout the process of formulating strategies to improve the adoption of care manager activities by PCNs, working group members took various contextual factors into account either explicitly or implicitly. From the thematic analysis of data, we were able to highlight four perspectives from which those contextual factors were considered: (1) the gap between patient needs and the accessibility of external mental health services; (2) the PCNs work environment and the gap between nursing activities and care manager activities; (3) The PCNs perception of their competencies and of their local leaders interest in supporting them; and (4) the perception of the ability of the FMG to implement change in nursing practices for people with CMDs.

#### 1) The gap between patient needs and the accessibility of external mental health services

In all three FMGs, clinicians decision to implement change in practices was influenced by their understanding of patients expectations and unmet needs regarding access and quality of mental health services. As reported by one PCN:

All too often, people arrive at the emergency room, or wherever, in a crisis, and they are sent home without any type of support or safety net. No one takes the time to tell them about the resources available to them. I often hear patients say: I went to the emergency room and they asked me if I was thinking about suicide? No. So they sent me home and didnt do a thing. (PCN09)

During problem analysis, local working groups were concerned about patient needs and asked the research teams about patients expectations. Working groups ultimately proposed strategies that they thought would contribute to improving the quality of patient follow-ups. Thus, working group participants considered their assessment of the gap between patient needs and the accessibility of external mental health services as a benchmark for the value of improving the role of PCNs for people with CMDs and long-term physical conditions.

#### 2) The PCNs work environment and the gap between nursing activities and care manager activities

Several characteristics of the PCNs work environment appeared to influence the formulation of strategies: the decision-making system surrounding nursing practices, the learning climate as it affected PCNs ability to spend time and energy learning new things, and the gap between nursing activities and care manager activities (i.e., the level of collaboration between team members, current nursing activities relating to patients with CMDs, and available resources in the FMG to deliver care for people with CMDs or other mental health problems).

The gap between current nursing practices and care manager activities had a particular influence over stakeholders perception of how much priority they should give to changing nursing practices. For instance, nurses in FMG02 had the most advanced practices compared to care manager activities, which was reflected by the research team to the working group. Consequently, their perception of the immediacy of the need to change their practices was lower than in the two other settings. The analysis of actual practices also helped local workings groups adopt a reflexive stance regarding their practices to identify specific areas for improvement and clarify problems that could be targeted with implementation strategies.

Furthermore, the way PCNs were involved in the decision-making process to improve their practices seemed to impact their assessment of the relative priority of implementing change. PCNs in FMG01 and FMG02 consulted each other during the project before changing their practice, and the results of this consultation was explicitly used in guiding decisions regarding strategy selection. By contrast, PCNs in FMG03 mentioned having little involvement in decision-making regarding the nature or the improvement of their practices, and they appeared less engaged in or concerned over the selection of strategies. As reported by a nurse in FMG03:

[] As I told you, we have many projects here. Just the Alzheimer project, the dementia project, took up a lot of time. Then, as we are aware, and its coming soon, very soon, there will be the COPD, [] its going to involve a lot of chronic patient follow-ups, adjustments, pumps, plus this, plus that. [] There is always a place for the integration of new [practices]. We [nurses] do not have a say, in the sense that it is not ourselves who decide if we integrate or retrieve something. We are consulted yes and no, I would say. (PCN13)

Additionally, the organizational climate surrounding learning new knowledge or skills seemed to influence the perceived value of implementing change in nursing practices. The learning climate seemed rather favorable for nurses at FMG01: they considered that their group dynamics encouraged learning new knowledge and skills to take care of people with CMDs, which they did not perceive to compete with other ongoing projects.

#### 3) The PCNs perception of their competencies and of their local leaders interest in supporting them

PCNs perception of their mental health care competencies (i.e., their knowledge and skills) appeared to have a considerable influence over their perception of a need for change and the strategies they were willing to prioritize. Several participants evoked their sense of self-efficacy regarding implementing change to justify how much they prioritized improving their practice. PCNs from FMG01 generally reported a good sense of self-efficacy when discussing implementing change, but the opposite was true in FMG03. Potential explanations for this discrepancy are the difference in formal support from the lead GP between FMGs and the ability of nurses in FMG01 to receive support from one of their peers who was already involved in the care of people with CMDs. It seemed that this person was perceived as a leader and a role model and that PCNs from FMG01 consequently had a growing interest in mental health:

Seven or eight years ago, when I started working in an FMG, my colleagues used to say Mental health makes me sick. [] Today, I have a colleague that I really love, she used to say I cant handle mental health. But now, she is the first to say that she would like to develop services for mental health It makes me happy to hear that, because I think our team is increasingly mature and that it has evolved a lot. Its the direction we are going toward. (PCN04)

#### 4) The perception of the ability of the FMG to implement the change in nursing practices for people with CMDs

Elements influencing the ability of FMGs to implement change constituted the main drivers for the discussion regarding the formulation and priorization of strategies (see ***Tables 4*** and ***5***). These elements were mainly related to the resources available to implement change (time, space, human resources), access to the materials or knowledge required to integrate care manager activities into the workflow of PCNs, and the engagement of a coalition of stakeholders in the implementation process. In other words, these elements were the contextual factors that would become the targets of specific implementation strategies.

#### Illustration of contextual factors influence on strategy formulation

**Figure 1:**
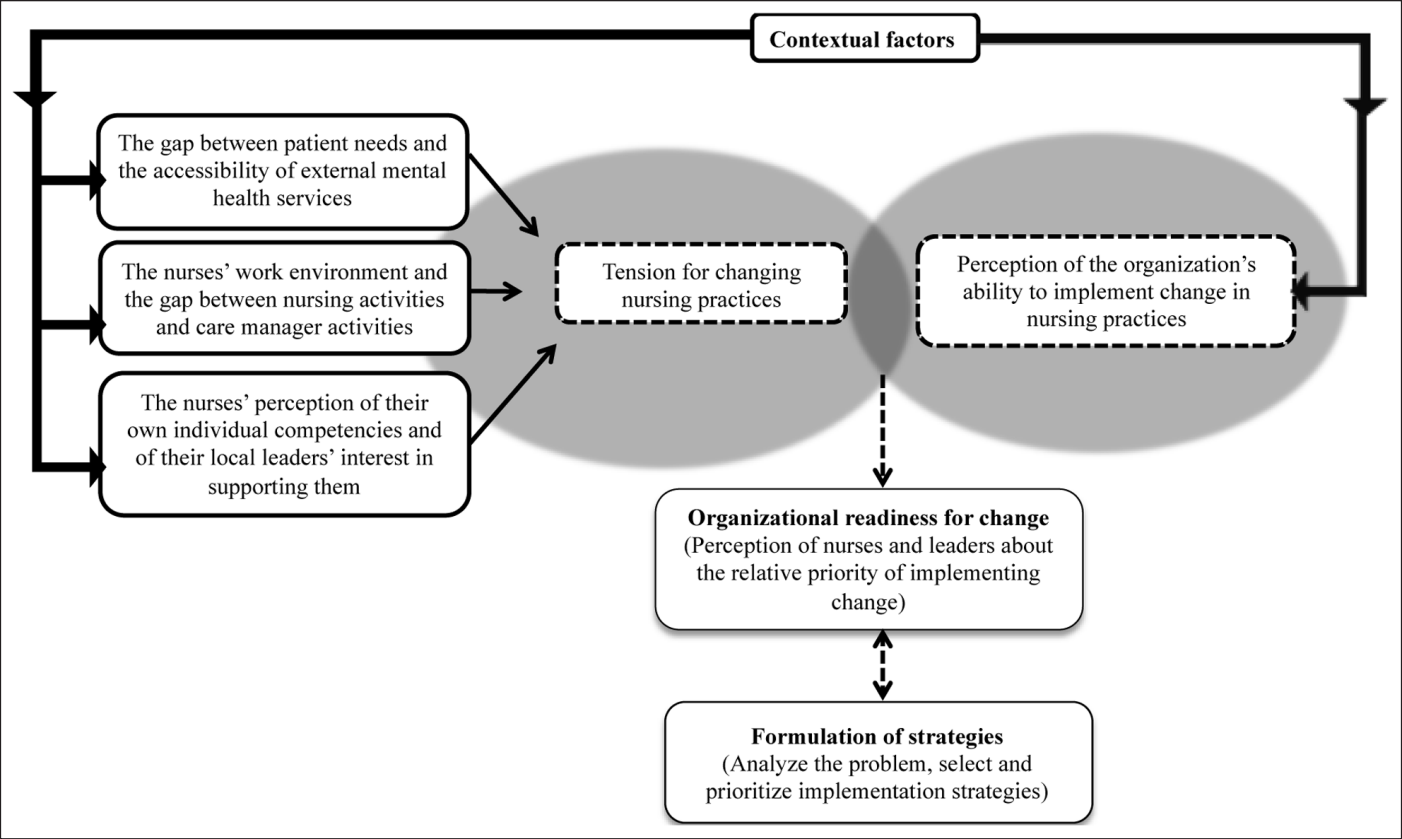
Influence of contextual factors on the formulation of implementation strategies to improve the adoption of care manager activities by primary care nurses.

The four perspectives through which stakeholders considered contextual factors appeared interconnected in their impact on strategy formulation. Specifically, the first three perspectives appeared to create tension for changing nursing practices. This tension for change refers to the way PCNs and leaders perceived the situation as intolerable and the value they gave to solving the problem. The tension for changing nursing practices thereafter interacted with the perceived ability of the FMG to implement possible strategies to improve the adoption of care manager activities by PCNs. Together, tension for change and the perceived organizational ability to implement change appeared to influence stakeholders perception of the relative priority of implementation, which we refer to as organizational readiness for change as proposed by Weiner (2009) [37]. Organizational readiness for change is conceptually different from the readiness for implementation construct in the CFIR [25], which is similar to the perceived ability of the FMG to implement strategies. Indeed, we found that it is through organizational readiness for change that contextual factors ultimately drive the overall process of formulating and prioritizing implementation strategies. The influence of contextual factors on the formulation of strategies through organizational readiness for change is illustrated in ***Figure 1***. Far from being static, organizational readiness for change evolved throughout implementation planning and strategy formulation.

## Discussion

This study is one of the first to analyze the influence of contextual factors on the formulation of implementation strategies to improve the adoption of care manager activities by PCNs. In this multiple case study, participants took into account contextual factors from four perspectives when considering potential strategies for implementation: (1) the gap between patient needs and the accessibility of external mental health services, (2) key characteristics of their work environment, (3) their assessment of their own individual skills and of their local leaders interest in supporting them, and (4) the ability they perceived in their organization to implement the change in practices they were considering. According to our analysis, those four perspectives were able to drive strategy formulation through organizational readiness for change, an evolving state that is distinct from the contextual factors themselves.

Using the CFIR, we were able to identify and label a broad range of contextual factors considered by participants when formulating strategies [25]. However, we did not find the CFIR to provide a sufficiently nuanced portrayal of the contextual factors influence on strategy formulation, which led us to use complementary theories to complete both intra-case and inter-case data analysis. Weiners proposition to consider organizational readiness for change as an intermediate step between contextual factors and the implementation of change [37] was key to further our understanding of how the implementation planning process may lead to actual improvement in care for patients with CMDs. Indeed, readiness for change is a fundamentally dynamic state that can be improved by providing support to organizations throughout the implementation process, particularly during the pre-implementation or implementation planning phase. Future studies should consider repeatedly assessing readiness for change as a potential indicator of the ongoing implementation process at the organizational level.

Our results help better understand how contextual factors or barriers and enablers identified in previous CCM implementation studies might have influenced the overall change process. Specifically, our results suggest that barriers to the implementation process may have dynamically influenced organizational readiness for change either by creating insufficient tension for changing collaborative practices (the perceived value of resolving the problem) or limiting the organizations perceived ability to implement change. Correspondingly, difficulty understanding the relative advantage of the model, lack of support from leaders, and perceived incompetence to manage care for people with CMDs are commonly mentioned barriers to implementing the CCM [18,19]. The presence of lead GPs interested in mental health is also recognized as facilitating the CCMs implementation [18,19,43]. In our study, interview participants were acutely aware of several of those barriers and enablers, which were at the forefront of local working groups discussions leading to strategy formulation. Encouragingly, this process allowed participating FMGs to find implementation strategies for which there was sufficiently high readiness for change in their organization, which would not have always been possible if we had only considered a set of pre-established implementation strategies. Supporting organizations in the formulation of implementation strategies appears to be a promising approach to contribute to improving the quality of care by identifying implementation strategies for which there is sufficient readiness for change. Future studies should further develop and evaluate interventions to support organizations in implementing evidence-based practices from a sound understanding of change theories [23].

### Strengths and limitations

An important strength of this study resides in its originality in investigating a critical phase of the implementation process, i.e., implementation planning, which has received insufficient attention to date. Studying implementation planning helps shed light on limitations in the scientific literature regarding effective methods to support implementation in primary healthcare organizations, especially by providing an accurate assessment of their actual professional practices. In turn, practices analysis allows stakeholders to understand the gap between their practices and evidence-based models such as the CCM, facilitating the mobilization of organizational readiness for change and the formulation of contextually appropriate strategies. Our original perspective allowed us to balance promoting CCM-oriented evidence-based practices and mobilizing organizational capacity for change through a bottom-up approach to strategy formulation. To support the validity of our results, we referred to credible models from the research literature throughout the research process: Grol & Wensings implementation of change model to contextualize how the research team supported stakeholders in implementation planning; the CFIR to understand the contextual factors that would be expected to influence strategy formulation; and recommendations issued from the ERIC study to categorize the implementation strategies that were considered and prioritized in each FMG. The use of those models will facilitate comparison with results from other studies in other contexts, including when research methodology may differ.

Despite its strengths, some limitations of this study should be highlighted. First, when evaluating actual practices, we could not entirely benefit from the same analysis techniques and tools as a more traditional implementation study. Indeed, this studys focus was not implementing the CCM or the role of care manager per se, but instead implementing strategies to enable clinicians to improve their practices and bring them closer to care management activities. When assessing actual practices, we did not work with stakeholders who explicitly understood their practices as CCM-related; the link between their practices and the model depended on the research teams accurate assessment of their activities. While this contributes to our results transferability to quality improvement initiatives beyond the field of implementation science, we may have missed or misinterpreted some relevant activities during analysis. Second, although we provide an overview of study participants and a detailed description of the context, including only three FMGs may have limited the results direct transferability even though it was necessary to allow for in-depth analysis of current practices feasibly. In future studies, adopting a more quantitative approach to assess and compare participating organizations contextual characteristics would be a convenient way to involve more cases and rigorously test the relationships between contextual factors and strategy formulation that we observed in this study. One way to achieve this would be to exploit clinical registries and existing mental health indicators [44]. Third, our open-ended approach to strategy formulation through integrated knowledge transfer has led individual FMGs to consider strategies that did not necessarily involve techniques and tools that have previously been validated or shown reliable in clinical trials. In that regard, our study shares some epistemological principles with participatory research, although more directive regarding the objective of adopting evidence-based practices belonging to the CCM. Fourth, the fact that the research project ended before the proposed strategies were actually implemented leaves several questions unanswered, most notably whether the strategies for which there was sufficient readiness for change were likely to produce the improvement in practices that was sought. Therefore, this study should not be seen as proposing an alternative to previously published CCM implementation studies, for example. Conducting the whole implementation process remains as crucial as ever. However, we believe that our results should convince stakeholders involved in future studies to pre-emptively include a well-structured approach to implementation planning.

## Conclusion

This study informs on the different perspectives from which to consider contextual factors when formulating implementation strategies related to the collaborative care model and the adoption of care manager activities by primary care nurses. Analyzing current practices, exploring contextual factors, and involving stakeholders in strategy formulation helped understand and mobilize clinicians readiness for change in each of the studys settings. While we did not test our implementation planning interventions effectiveness on organizational or clinical outcomes, we encourage other researchers to take our results into account when designing implementation studies. Including a well-designed implementation planning phase in future implementation studies should further advance knowledge on ways to strategically implement evidence-based interventions for patients with common mental disorders and long-term physical conditions in primary care settings.

## Additional Files

The additional files for this article can be found as follows:

10.5334/ijic.5556.s1 Additional File 1. Analysis table to assess the quality and the level of achievement of actual collaborative care activities for people with common mental disorders and physical long-term conditions.

10.5334/ijic.5556.s2 Additional File 2. Analysis table to assess the quality and the level of achievement of actual primary care nurses activities in care management of people with common mental disorders (CMDs) and physical long-term conditions (LTCs).

## References

[1] Public Health Agency of Canada. Prevalence of Chronic Diseases Among Canadian Adult. [webpage on the internet]. [cited 2020 11 June]; updated 2019 December 09]. Available from: https://www.canada.ca/en/public-health/services/chronic-diseases/prevalence-canadian-adults-infographic-2019.html.

[2] Cassell A, Edwards D, Harshfield A, Rhodes K, Brimicombe J, Payne R, Griffin S. The epidemiology of multimorbidity in primary care: a retrospective cohort study. British Journal of General Practice, 2018; 68: e245 51. DOI: 10.3399/bjgp18X695465 PMC5863678 29530918

[3] Teesson M, Mitchell PB, Deady M, Memedovic S, Slade T, Baillie A. Affective and Anxiety Disorders and their Relationship with Chronic Physical Conditions in Australia: Findings of the 2007 National Survey of Mental Health and Wellbeing. Australian & New Zealand Journal of Psychiatry, 2011; 45(11): 939 46. DOI: 10.3109/00048674.2011.614590 21967412

[4] Vogeli C, Shields AE, Lee TA, Gibson TB, Marder WD, Weiss KB, Blumenthal D. Multiple chronic conditions: Prevalence, health consequences, and implications for quality, care management, and costs. Journal of general internal medicine, 2007; 22(3): 391 95. DOI: 10.1007/s11606-007-0322-1 18026807 PMC2150598

[5] Gaulin M, Simard M, Candas B, Lesage A, Sirois C. Combined impacts of multimorbidity and mental disorders on frequent emergency department visits: A retrospective cohort study in Quebec, Canada. Canadian Medical Association Journal, 2019; 191(26): E724 32. DOI: 10.1503/cmaj.181712 31266786 PMC6606417

[6] Katon W, Von Korff M, Lin E, Walker E, Simon GE, Bush T, Robinson P, Russo J. Collaborative management to achieve treatment guidelines: Impact on depression in primary care. JAMA, 1995; 273(12): 1026 31. DOI: 10.1001/jama.1995.03520370068039 7897786

[7] American Psychiatric Association/Academy of Psychosomatic Medicine. Dissemination of integrated care within adult primary care settings. The Collaborative Care Model; 2016 4. [cited 2020 11 Jun]. Available from: https://www.psychiatry.org/psychiatrists/practice/professional-interests/integrated-care/learn.

[8] Archer J, Bower P, Gilbody S, Lovell K, Richards D, Gask L, Dickens C, Coventry P. Collaborative care for depression and anxiety problems (Review). Cochrane Database Systematic Reviews, 2012; 10: 1 277. DOI: 10.1002/14651858.CD006525.pub2 PMC11627142 23076925

[9] Katon WJ, Elizabeth HB, Von Korff M, Ciechanowski P, Ludman EJ, Young B, Peterson D, Rutter CM, McGregor M, McCulloch D. Collaborative care for patients with depression and chronic Illnesses. The new England journal of Medicine, 2010; 363(27): 2611 20. DOI: 10.1056/NEJMoa1003955 21190455 PMC3312811

[10] Gilbody S, Bower P, Whitty P. Costs and Consequences of enhanced primary care for depression (Systematic review of randomized economic evaluations). British Journal of Psychiatry, 2006; 189: 297 308. DOI: 10.1192/bjp.bp.105.016006 17012652

[11] University of Washington AIMS Center. CoCM Behavioral Health Care Manager: Sample Job Description, Typical Workload & Resources Requirements; 2017. [cited 2020 11 Jun]. Available from: http://aims.uw.edu/sites/default/files/CareManagerJobDescription_0.pdf.

[12] Ekers D, Murphy R, Archer J, Ebenezer C, Kemp D, Gilbody S. Nurse-delivered collaborative care for depression and long-term physical conditions: A systematic review and meta-analysis. Journal of Affective Disorders, 2013; 149(13): 14 22. DOI: 10.1016/j.jad.2013.02.032 23545062

[13] Askerud A, Conder J. Patients experiences of nurse case management in primary care: A meta-synthesis. Australian Journal of Primary Health, 2017; 23(5): 420 428. DOI: 10.1071/PY17040 28923163

[14] Adams, EG. Treatment of depression in Integrated care: Implementation of the Nurse care Manager. SAGE Open Nursing, 2019; 5: 1 9. DOI: 10.1177/2377960819861862 PMC7774405 33415247

[15] Girard A, Hudon C, Poitras ME, Roberge P, Chouinard MC. Primary care nursing activities with patients affected by physical chronic disease and common mental disorders: A qualitative descriptive study. Journal of Clinical Nursing, 2017; 26(910): 1385 1394. DOI: 10.1111/jocn.13695 28000321

[16] Overbeck G, Kousgaard MB, Davidsen AS. The work and challenges of care managers in the implementation of collaborative care: A qualitative study. Journal of Psychiatric and Mental Health Nursing, 2018; 25(3): 167 175. DOI: 10.1111/jpm.12449 29283474

[17] Girard A, Ellefsen , Roberge P, Carrier JD, Hudon C. Challenges of adopting the role of care manager when implementing the collaborative care model for people with common mental illnesses: A scoping review. International Journal of Mental Health Nursing, 2019; 28(2): 369 389. DOI: 10.1111/inm.12584 30815993

[18] Wood E, Ohlsen S, Ricketts T. What are the barriers and facilitators to implementing Collaborative Care for depression? A systematic review. Journal of Affective Disorders, 2017; 214: 26 43. DOI: 10.1016/j.jad.2017.02.028 28266319

[19] Overbeck G, Davidsen AS, Kousgaard MB. Enablers and barriers to implementing collaborative care for anxiety and depression: A systematic qualitative review. Implementation Science, 2016; 11(165): 1 16. DOI: 10.1186/s13012-016-0519-y 28031028 PMC5192575

[20] Proctor EK, Powell BJ, McMillen JC. Implementation strategies: Recommendations for specifying and reporting. Implementation Science, 2013; 8(139): 1 11. DOI: 10.1186/1748-5908-8-139 24289295 PMC3882890

[21] Miler CJ, Sullivan JL, Kim B, Elwy AR, Drummond KL, Connolly S, Riendeau RP, Bauer MS. Assessing collaborative care in mental health teams: Qualitative analysis to guide future. Administration and Policy in Mental health and Mental Health Services Research, 2019; 46(2): 154 166. DOI: 10.1007/s10488-018-0901-y 30353419

[22] Chen HT. Practical program evaluation: Theory-driven evaluation and the integrated evaluation perspective, 2^nd^ ed. Los Angeles, CA: Sage Publications; 2015. 35 57.

[23] Waltz TJ, Powell BJ, Fernandez ME, Abadie B, Damschroder LJ. Choosing implementation strategies to address contextual barriers: Diversity in recommendations and future directions. Implementation Science, 2019; 14(42): 1 15. DOI: 10.1186/s13012-019-0892-4 31036028 PMC6489173

[24] Leeman J, Birken SA, Powell BJ, Rohweder C, Shea MC. Beyond implementation strategies: Classifying the full range of strategies used in implementation science and practice. Implementation Science, 2017; 12(125): 1 9. DOI: 10.1186/s13012-017-0657-x 29100551 PMC5670723

[25] Damschroder LJ, Aron DC, Keith RE, Kirsh SR, Alexander JA, Lowery JC. Fostering implementation of health services research findings into practice: A consolidated framework for advancing implementation science. Implementation Science, 2009; 4(50): 1 15. DOI: 10.1186/1748-5908-4-50 19664226 PMC2736161

[26] Powell BJ, Beidas RS, Lewis CC, Aarons GA, McMillen JC, Proctor EK, Mandell DS. Methods to Improve the Selection and Tailoring of Implementation Strategies. The Journal of Behavioral Health Services & Research, 2017; 44(2): 177 194. DOI: 10.1007/s11414-015-9475-6 26289563 PMC4761530

[27] Ministre de la sant et des services sociaux. Programme de financement et de soutien professionnel pour les groupes de mdecine de famille [Funding and Professional Support Program for Family Medicine Groups]; 2017 6. [cited 2020 Jun]. Available from: https://publications.msss.gouv.qc.ca/msss/fichiers/2017/17-920-09W.pdf [in French].

[28] Ministre de la sant et des services sociaux. Guide pratique lintention des infirmires cliniciennes qui travaillent dans un groupe de mdecine de famille ou un groupe de mdecine de famille universitaire. [A practical guide for registered nurse working in a family medicine group or a university family medicine group]; 2019. [cited 2020 Jun]. Available from: https://publications.msss.gouv.qc.ca/msss/fichiers/2019/19-924-11W.pdf [in French].

[29] Merriam SB. Qualitative research and case study applications in education, 2^nd^ ed. San Francisco, CA: Jossey-Bass Publishers; 1998.

[30] Merriam SB, Tisdell EJ. Qualitative Research: A Guide to Design and Implementation, 4^th^ ed. San Francisco, CA: Jossey-Bass Publishers; 2016.

[31] Gagliardi AR, Whitney B, Khotari A, Boyko J, Urquhart R. Integrated knowledge translation (IKT) in health care: A scoping review. Implementation science, 2016; 11(38): 1 12. DOI: 10.1186/s13012-016-0399-1 26988000 PMC4797171

[32] Grol R, Wensing M. Effective implementation of change in healthcare: A systematic approach. In Improving Patient Care: The Implementation of Change in Health Care, 2^nd^ ed. Hoboken, NJ: John Wiley & Sons; 2013. DOI: 10.1002/9781118525975.ch3

[33] Baker GR. The contribution of case study research to knowledge of how to improve quality of care. BMJ Quality & Safety, 2011; 20: i30 35. DOI: 10.1136/bmjqs.2010.046490 PMC3066793 21450767

[34] Roberge P, Hudon C, Pavilanis A, Beaulieu MC, Benot A, Brouillet H, Vanasse A. A qualitative study of perceived needs and factors associated with the quality of care common mental disorders in patients with chronic diseases: the perspective of primary care clinicians and patients. BMC Family Practice, 2016; 17(134): 1 14. DOI: 10.1186/s12875-016-0531-y 27620166 PMC5020556

[35] University of Washington AIMS Center. Patient-Centered Integrated Behavioral Health Care Principles & Tasks Checklist; 2014. [cited 2020 11 Jun]. Available from: https://aims.uw.edu/sites/default/files/CollaborativeCarePrinciplesAndComponents_2014-12-23.pdf.

[36] Miles MB, Huberman AM, Saldana J. Qualitative Data Analysis: A methods Sourcebook, 3^rd^ ed. Thousand Oaks, CA: SAGE Publications; 2014.

[37] Weiner B. A theory of organizational readiness for change. Implementation Science, 2009; 4(67): 1 9. DOI: 10.1186/1748-5908-4-67 19840381 PMC2770024

[38] Girard A. Study of the implementation planning process favoring the role of care manager by primary care nurses for people with common mental disorders and long-term physical condition [doctoral thesis], Universit de Sherbrooke, 2020.

[39] Poitras ME, Fortin M, Hudon C, Haggerty J, Almirall J. Validation of the disease burden morbidity assessment by self-report in a French-speaking population. BMC Health Services Research, 2012; 12(35): 2 6. DOI: 10.1186/1472-6963-12-35 22333434 PMC3305524

[40] Bayliss EA, Ellis JL, Steiner JF. Subjective assessments of comorbidity correlate with quality of life health outcomes: Initial validation of a comorbidity assessment instrument. Health and Quality of Life Outcomes, 2005; 3(51): 1 8. DOI: 10.1186/1477-7525-3-51 16137329 PMC1208932

[41] Powell BJ, Waltz TJ, Chinman MJ, Damschroder LJ, Smith JL, Matthieu MM, Proctor EK, Kirchner JE. A refined compilation of implementation strategies: Results from the Expert Recommandations for Implementing Change (ERIC) project. Implementation Science, 2015; 10(21): 1 14. DOI: 10.1186/s13012-015-0209-1 25889199 PMC4328074

[42] Waltz TJ, Powell BJ, Matthieu MM, Damschroder LJ, Chinman MJ, Smith JL, Proctor EK, Kirchner JE. Use of concept mapping to characterize relationships among implementation strategies and assess their feasibility and importance: Results from the Expert Recommendations for Implementing Change (ERIC) study. Implementation Science, 2015; 10(109); 1 8. DOI: 10.1186/s13012-015-0295-0 26249843 PMC4527340

[43] Sunderji N, Ion A, Zhu A, Perivolaris A, Rodie D, Mulsant BH. Challenges in conducting research on collaborative health care: a qualitative study. CMAJ Open, 2019; 7(2); E405 14. DOI: 10.9778/cmajo.20180172 PMC6579651 31201177

[44] Moise N, Shah RN, Essock S, Jones A, Carruthers J, Handley MA, Peccoralo L, Sedere L. Sustainability of collaborative care management for depression in primary care settings with academic affiliations across New York State. Implementation Science, 2018; 13(128): 1 17. DOI: 10.1186/s13012-018-0818-6 30314522 PMC6186053

