# Supplementary material for: The Influence of Contextual Factors on the Process of Formulating Strategies to Improve the Adoption of Care Manager Activities by Primary Care Nurses
Source: Int J Integr Care. 2021 May 19;21(2):20. doi: 10.5334/ijic.5556 (PMC8139289; doi:10.5334/ijic.5556)
Supplement: Additional File 1. — Analysis table to assess the quality and the level of achievement of actual collaborative care activities for people with common mental disorders and physical long-term conditions. [file ijic-21-2-5556-s1.pdf]

Additional file 1

Analysis table to assess the quality and the level of achievement of actual collaborative care activities for people with common mental disorders and physical long-term conditions

(Adapted from "Patient-Centered Integrated Behavioral Health Care Principles & Tasks Checklist"-AIMS Center, University of Washington, 2014)[1]

Professional activities of the collaborative care model	Does not seem to be done	Sometimes done	Done	By Whom & How
1. Detection of symptoms (M), medical evaluation (N) and diagnosis				
Screen for anxiety or depressive disorder or other mental health problems for people with physical LTCs (M) using valid measurement tools				
Review of relevant laboratory and clinical parameters (N) *				
Diagnosis of anxiety or depressive disorder (M) and related conditions following a medical evaluation (N)				
Use of valid measurement tools to assess and document baseline symptom severity				
2. Engagement and support of patient and family in care (M)				
Introduce patient to collaborative care team (M)				
Explain the care patients will receive for their conditions (N)				
Value patients and families lived expertise and advocacy ** (N)				
Care is responsive to patient needs and preferences ** (N)				

[1] Used with permission from the University of Washington AIMS Center, [November 2019]

Initiate patient tracking in population-based registry				
3. Develop and provide an evidence-based treatment				
Develop and regularly update a biopsychosocial treatment plan according to patients'needs and preferences (M)				
Prescribe and manage medications for anxiety or depressive disorders and for physical LTCs (M) as clinically indicated				
Provide evidence-based psychotherapy (e.g. cognitive behavioral therapy, problem-solving treatment, interpersonal therapy)				
Change or adjust treatments if patients do not meet treatment targets				
Educate patients and family about symptoms and treatments (M)				
Provide education on self-management skills to patients and their families (M)				
Provide evidence-based counseling (e.g. motivational interviewing, behavioral activation)				
4. Systematically follow up, adjust treatment and develop a relapse prevention plan				
Use population-based registry to systematically follow all patients				
Identify patients who are not improving to target them for psychiatric consultation and treatment adjustment				
Proactively reach out to patients who do not follow-up				
Monitor treatment side effects and complications				
Monitor treatment response at each contact with valid outcome measures				
Create and support relapse prevention plan when patients are substantially improved				
5. Communication and care coordination				
Coordinate and facilitate effective communication among providers				
Engage and support family and significant others as clinically appropriate				
Facilitate referrals to specialty care, social services and community-based resources (M)				
Track referrals to specialty services, social services and community services (M)				

6. Systematic psychiatrist case review and consultation				
Provide psychiatric assessments in person or via telemedicine for patients with complex needs that can create challenges for the team (M)				
Conduct regular (e.g. weekly) psychiatric caseload review on patients who are not improving				
Provide specific recommendations for additional diagnostic work up, treatment changes, or referrals				
7. Collaborative care oversight (M) and quality improvement				
Provide clinical support and supervision for care and services (M)				
Provide administrative support and supervision for care and services (M)				
Routinely examine provider- and collaborative care-level outcomes (e.g. clinical outcomes, quality of care, patient satisfaction) and use this information for quality improvement				

(M) Modified (N) New component

*** Retrieved from COMPASS Intervention Guide:** The COMPASS consortium. (2015). The Care of Mental, Physical and Substance Use Syndromes Intervention Guide. Retrieve from https://www.icsi.org/wp-content/uploads/2019/08/COMPASSInterventionGuide082019.pdf

**** Retrieved from QI4CC framework:** Sunderji N, Ghavam-Rassoul A, Allyson I, Elizabeth L. Driving improvements in the implementation of collaborative mental health care: A Quality framework to guide measurement, improvement and research. Toronto, Canada; 2016.
