# Supplementary material for: The Influence of Contextual Factors on the Process of Formulating Strategies to Improve the Adoption of Care Manager Activities by Primary Care Nurses
Source: Int J Integr Care. 2021 May 19;21(2):20. doi: 10.5334/ijic.5556 (PMC8139289; doi:10.5334/ijic.5556)
Supplement: Additional File 2. — Analysis table to assess the quality and the level of achievement of actual primary care nurses activities in care management of people with common mental disorders (CMDs) and physical long-term conditions (LTCs). [file ijic-21-2-5556-s2.pdf]

Additional file 2

Analysis table to assess the quality and the level of achievement of actual primary care nurses' activities in care management of people with common mental disorders (CMDs) and physical long-term conditions (LTCs)

Care manager's activities for people with CMDs and LTCs	Does not seem to be done	Sometimes done	Done	Not done	Competencies (skills, knowledge, attitude or behaviour)	Barriers and enablers
Coordinate care between service providers (private or public psychosocial services, community organizations, addiction rehabilitation centre specialized services [e.g. psychiatry] other hospital centre services) and the multidisciplinary team (social worker, psychologist, psychiatrist, family doctor, pharmacist).						
Assess global health condition (e.g. mood, lifestyle, level of functioning, etc.).						
Screen for anxious and depressive disorders using validate tools						
Participate in meetings with professionals (family doctor or mental health specialist) to provide information about patient progress.						
Document patient progress and treatment recommendations in electronic health record.						
Track patient follow-up and clinical outcomes using a patient registry.						
Document in-person and telephone encounters in the registry and use the system to identify and re-engage						

patients.						
Facilitate patient engagement and follow-up care.						
Systematically monitor symptoms and response to psychotherapeutic and pharmacological treatments (in person or over the phone).						
Provide brief psychosocial interventions using evidence-based techniques such as behavioral activation, motivational interviewing, cognitive-behavioral strategies and problem solving.						
Provide education to the patient and their family about mental disorders (including substance use disorders) and available treatments.						
Support the patient in the management of medications prescribed for CMDs and LTCs by evaluating adherence to treatment, side effects and effectiveness of medications.						
Facilitate in-clinic or outside referrals to evidence-based psychotherapeutic treatments as clinically indicated.						
Facilitate referrals for clinically indicated services outside of the clinic (e.g. social services, rehabilitation services, specialized mental health services, addiction services).						
Regularly review records with a consultant psychiatrist or other mental health specialist and indicate any necessary changes to the file.						
Contribute to the modification of the treatment plan for patients who do not improve as expected in collaboration with the family doctor and the consultant psychiatrist.						
Develop and complete a relapse prevention plan with patients who have achieved their treatment goals.						

Additional files-Analysis tables

Data sources: "CoCM Behavioral Health Care Manager: Sample Job Description, Typical Workload & Resource Requirements"-AIMS Center, University of Washington, 2017[1].

[1] Used with permission from the University of Washington AIMS Center, [November 2019]
